# Ergo-Nutritional Intervention in Basketball: A Systematic Review

**DOI:** 10.3390/nu14030638

**Published:** 2022-02-02

**Authors:** Ignacio Escribano-Ott, Julio Calleja-González, Juan Mielgo-Ayuso

**Affiliations:** 1 Department of Physical Education and Sport, Faculty of Education and Sport, University of the Basque Country, 01007 Vitoria, Spain; julio.calleja@ehu.eus; 2 Department of Health Sciences, Faculty of Health Sciences, University of Burgos, 09001 Burgos, Spain; jfmielgo@ubu.es

**Keywords:** basketball, supplement, ergo-nutritional aid, caffeine, creatine, vitamin D, recovery, performance

## Abstract

Using nutritional supplements is a widespread strategy among basketball players to ensure the appropriate provision of energy and nutrients to avoid certain complaints. Particularly in basketball, there is no consensus on the type, quantity or form of use in which these supplements should be administered. Therefore, the main aim of this systematic review is to highlight the ergo-nutritional aids that may be effective in basketball. A structured search was carried out following the Preferred Reporting Items for Systematic Review and Meta-Analyses (PRISMA^®^) guidelines in the Medline/PubMed and Web of Science, Cochrane Library, and Scopus databases until 31 December 2021; no year restriction was applied to the search strategy. There were no filters applied to the basketball players’ level, gender, race, or age to increase the power of the analysis. The results of this systematic review have shown that the effective dose of caffeine to enhance anaerobic performance and the feeling of vigorousness and energy ranges from 3 to 6 mg·kg^−1^, showing more positive effects when is supplemented 60–75 min before exercise in the morning and in test-based task. On the other hand, vitamin E (ranging from 200 to 268 mg), vitamin D (10,000 IU) and EPA (2 g) may have a potential role in recovery and wellness. The primary limitation of this study is the scarcity of studies related to nutritional supplementation in basketball players. However, a major strength is that this is the first systematic review describing what ergo-nutritional aids may be specifically helpful for basketball. Despite the need for future studies, certain nutritional supplements may have promising advantages for basketball (long-term supplementation of nitrates for recovery), whereas others (β-alanine, sodium bicarbonate, and acute nitrate supplementation) might theoretically be regarded as not interesting for basketball, or even not recommended by the World Anti-Doping Agency (WADA) as bovine colostrum.

## 1. Introduction

Basketball is one of the most popular sports in the world, played by males and females of all levels and ages [1]. When a player achieves elite performance, they can play in the highest national, international, continental, and world championships and Olympic Basketball Games [1]. Undoubtedly, players must meet high physical, physiological and psychological demands [2,3]. During a basketball game, a player can perform more than 2000 explosive actions, reaching 85% of their maximum heart rate and accumulating concentrations of lactate in the blood from 2.7 to 6.8 mmol [1,2,3]. In addition to high-intensity actions, basketball players must also have a suitable aerobic form (VO_2 max_ in the range 50–60 mLO_2_/Kg/min) to cope with the high-intensity efforts required (4–5 km covered on average) and recover between games and training sessions. [1,2,3]. In addition, strength and force production, agility and speed are also key components of good performance, as they are required for numerous basketball movements. [1]. Thus, ATP-CP and glycolysis pathways play an important role in energy production [1]. Furthermore, basketball players must complete their recovery (energy, structure, psychological and functional) quickly and effectively because they have little time to recover (due to the large number of games in one full season and many hours of travel) [4,5,6,7]. Incorrect recovery can result in high levels of fatigue which negatively affect the player’s performance and may increase the risk of injury. [5]. Thus, basketball teams are highly engaged in implementing recovery strategies [5], and good nutrition plays a key role as one such strategy. [8]. 

Proper nutrition helps maintain physical and cognitive performance [9,10,11,12,13], supports injury prevention, the return-to-play process, training adaptations [14] and finally accelerate and optimize recovery processes [15]. To achieve this, a nutritional plan must be based on the uniqueness of individuals [16], the playing position [17], the workload [18] and other logistics factors such as travel [19] or food preferences [20]. Generally, basketball players are big athletes [21] with a high muscle mass [22,23], so they have high energy needs that involve eating large quantities of food. Therefore, to avoid gastrointestinal discomfort [9], they should consider splitting the high volume of food they must eat [24].

In summary, basketball players must be very careful about the timing, type and amount of nutrients they must take from their diet [8]. This practical complexity usually drives basketball players to use ergo-nutritional aids to ease the achievement of their nutritional goals [25,26,27]. However, there are numerous ergo-nutritional supplements in the market, and around them, studies of dubious quality (doubtful methodologies, non-standardized protocols, and publication bias) [28,29]. Although some of these supplements have been scientifically proven to be secure and have a potential ergogenic effect [30] in specific sports [31,32,33], to the best of the author’s knowledge, there is no previous systematic analysis that collects all the ergogenic-nutritional aids that may be of interest to cope the basketball game/practice demands or help on the player’s recovery process.

In fact, a widespread variety of ergo-nutritional supplements have been analyzed in basketball, but in many instances, these findings have been found to be controversial or contradictory. For example, sports drinks are a well-known source of water, carbohydrates (CHO) and electrolytes to prevent or treat dehydration [34]. However, the formulation of this beverage varies from 7.2% sugar, 0.8% maltodextrins, and 510 mg/L Na to 6% CHO and 18.0 mM of Na among studies [35,36,37]. Regarding vitamins (VIT) or multivitamins, athletes are really keen to consume them under the premise that they would improve their health and enhance performance or recovery [38,39,40], but interventions confirming this theory are nevertheless scarce, with only one carried out with a strong scientific evidence such is VIT D [41]. Finally, a very popular ergo-nutritional aid is caffeine (CAF). The administration of 3 mg·kg^−1^ has shown small-moderate [42] to strong [43] effects over anaerobic performance, whereas in other works to reach these achievements a dosage of 6 mg·kg-^1^ was necessary [44,45,46]. With respect to protein supplementation, although many studies used ranges from 20 to 25 g, the source of protein (PRO) (whey, casein, and bovine colostrum) varied among studies [47,48,49]. The form of use is another controversial factor that can lead to different results, such as in the case of nitrate supplementation (NIT), with positive results when administered in meals [50] but negative when used in beverages [51].

Therefore, in the face of this lack of paradigm, this systematic review aims to gather and organize those ergo-nutritional aids that have scientifically proven their effectiveness in basketball to enhance on-court performance and recovery, offering players, coaches and teams a final document to help them make their selection.

## 2. Materials and Methods

### 2.1. Search Strategy and Study Selection

The main aim of this systematic review is to highlight the ergo-nutritional aids that may be effective in basketball. It was carried out following the Preferred Reporting Items for Systematic Reviews and Meta-Analyses (PRISMA^®^) guidelines, which helped to improve the integrity of this systematic review [52]. The PICOS model was used to determine the inclusion criteria [53]—P (Population): “basketball players”; I (Intervention): “ergo-nutritional supplementation”; C (Comparators): “same conditions with placebo”; O (Outcome): “any parameter related to basketball practice”; and S (study design): “double-blind and randomized design, reviews, metanalysis, study cases, descriptive and pre-post intervention” [54]. Before the search, a review protocol based on PRISMA-P^®^ [52] was completed and registered at PROSPERO (ID = 274395). The review protocol was updated during the review process and is available at https://www.crd.york.ac.uk/PROSPEROFILES/274395_STRATEGY_20220103.pdf (last accessed 3 January 2022).

A structured search was conducted in the following databases: PubMed/MEDLINE, Web of Science (WOS), Cochrane Library, and Scopus. It included results until 31 December 2021, while no year restriction was applied to the search strategy. Search terms included a mix of medical subject headings (MeSH) and free-text words for key concepts related to ergo-nutritional supplementation and basketball performance. Specifically, the following search equation was used: Basketball [MeSH] OR Nutritional Supplements [MeSH] OR Ergo nutritional aids [MeSH] OR Dietary Supplements [MeSH] OR Athletic Performance [MeSH] AND Basketball NOT “wheelchair” AND (“physical performance” [All Fields] OR “physical endurance” [All Fields] OR “physical” [All Fields] OR “endurance” [All Fields] OR “performance” [All Fields] OR “aerobic” [All Fields] OR “anaerobic” [All Fields] OR “body composition” [All Fields] OR “(anthropo*” [All Fields]) AND (“nutri*” [All Fields]) OR “supplementation” AND “basketball”, which returned relevant articles applying the snowball strategy [55]. All titles and abstracts from the search were cross-referenced to identify duplicates and any potential missing studies. Titles and abstracts were screened for a subsequent full-text review. The search for published studies was independently performed by two different authors (I.E.-O. and J.C.-G.) and disagreements were resolved through discussions with a third one (J.M.-A.).

### 2.2. Inclusion and Exclusion Criteria

#### 2.2.1. Inclusion Criteria

There were no filters applied to the basketball players’ level, race, gender, and position on the court or age to increase the power of the analysis. However, for the articles obtained in the database search, the following inclusion criteria were applied to select the final studies: (I) an experimental condition that included the ingestion of an ergo-nutritional supplement before and/or during exercise, which was compared to an identical experimental condition with the ingestion of a placebo; (II) testing the effects of an ergo-nutritional supplement on basketball-specific skills, tests and/or real or simulated matches; (III) with and/or without a blinded and randomized design; (IV) with clear information regarding the administration of supplementation (relative dose of the ergo-nutritional supplement per kg of body mass and/or absolute dose of the ergo-nutritional supplement with information about body mass, timing of intake); (V) published in any language; (VI) conducted specifically with basketball players.

#### 2.2.2. Exclusion Criteria

On the other hand, the following exclusion criteria were applied to the experimental protocols of the investigation: (I) studies that were not conducted on basketball players; (II) studies that were performed for clinical purposes or therapeutic use; (III) studies carried out using participants with a previous medical condition, illness, special populations, drugs or injured athletes.

### 2.3. Variable Outcomes

Primary outcomes and data on outcome measure reporting were systematically extracted and categorized. The choice of the most suitable outcome was based on the previous research question. Thus, our systematic review focus on the following athlete-centered primary outcomes: changes in muscle strength, sprint, agility, jump, skills and cognitive performance; changes in body composition; aerobic and anaerobic performance; antioxidant status, oxidative stress, hormonal status, pro-inflammatory and anti-inflammatory markers; vitamin status; adverse side effects and subjective perceptions. These values were obtained from different tests. For the secondary outcomes, changes in blood lactate concentration, glucose levels, heart rate and changes in urine variables were considered.

### 2.4. Data Extraction

Once the inclusion/exclusion criteria were applied to each selected study, the following data were extracted by two authors (I.E.-O. and J.C.-G.) using a spreadsheet: authors and year of publication, sample size, level and gender, study design, type of protocol, type of nutritional supplement, relative and absolute dose, primary and secondary outcomes. Outcomes were clustered by the effect of the intervention on aerobic, anaerobic and skill performance, body composition, wellness and recovery. Subsequently, disagreements were resolved through discussion until a consensus was achieved with the third one (J.M.-A.).

### 2.5. Quality Assessment of the Experiments: Risk of Bias

**Figure 1:**
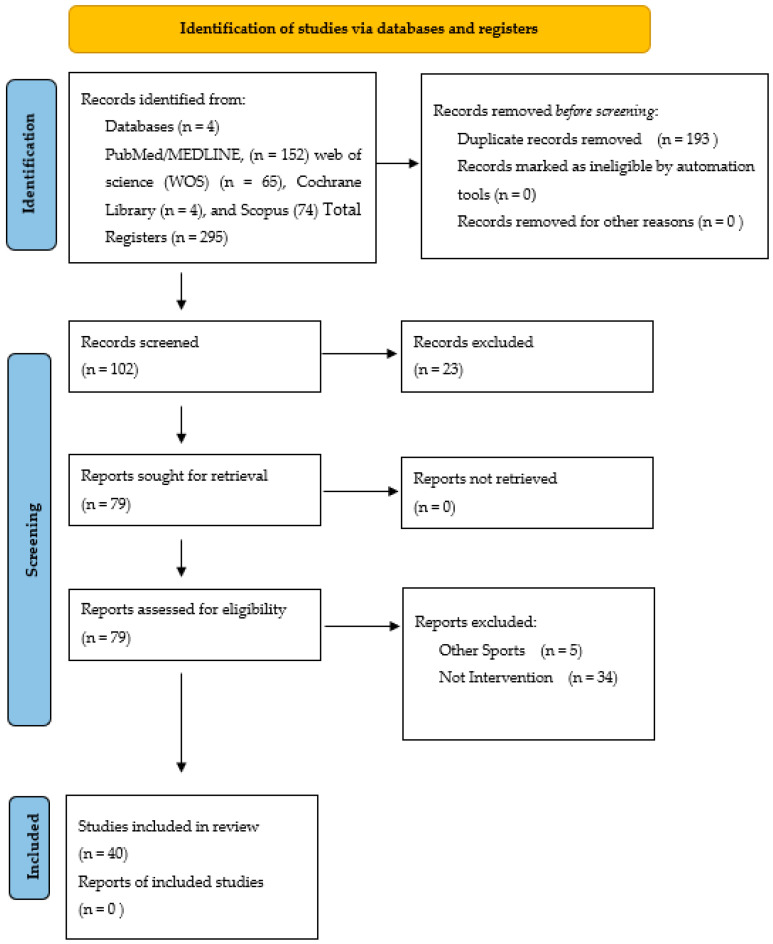
Selection of studies.

**Figure 2:**
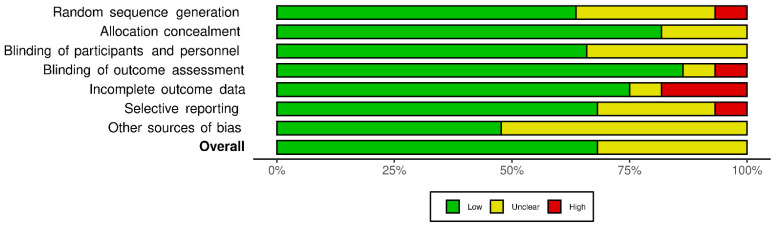
Risk of bias summary: review authors’ judgements about each risk of bias item for each included study.

**Figure 3:**
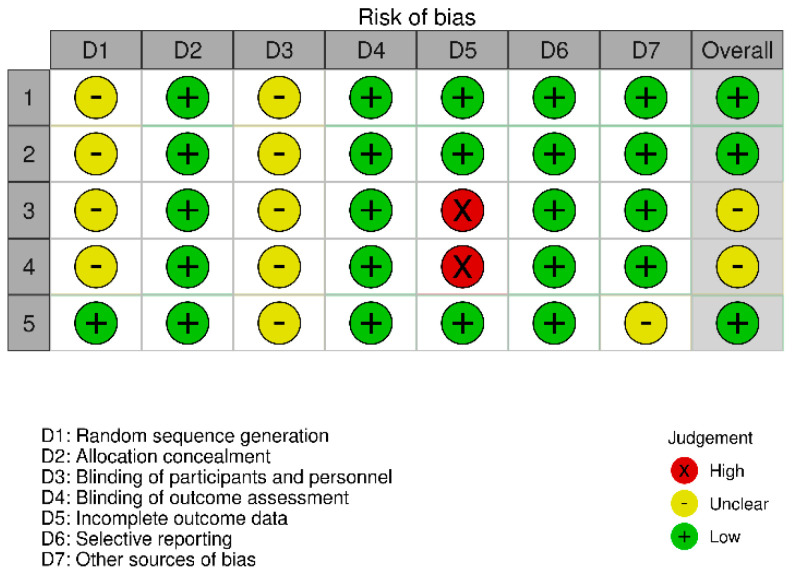
Risk of bias domains traffic-light plot.

**Figure 4:**
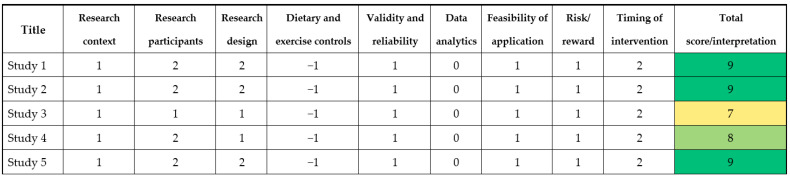
From paper to podium matrix.

Methodological quality and risk of bias were assessed by two authors independently (I.E.-O. and J.C.-G.), and disagreements were resolved by a third-party evaluation (J.M.-A.), in accordance with the Cochrane Collaboration Guidelines [54]. The items on the list were divided into different domains: random sequence generation (selection bias), allocation concealment (selection bias), blinding of participants and personnel (performance bias), blinding of outcome assessment (detection bias), incomplete outcome data (attrition bias), selective reporting (reporting bias), and other types of bias. They were characterized as “low” if criteria for a low risk of bias were met (plausible bias unlikely to seriously alter the results) or “high” if criteria for a high risk of bias were met (plausible bias that seriously weakens confidence in the results). If the risk of bias was unknown, it was considered “unclear” (plausible bias that raises some doubts about the results). [54]. The full details are given in Figure 1, Figure 2 and Figure 3. Studies that used two different dosages, more than one supplement or a combination of them were treated as independent works. Most of the trials assessed showed an unclear level in the criteria of other types of bias due to incomplete reporting or monitoring of not controlled but relevant variables such as diet or meal pattern description. The robustness and real-world application of the ergo-nutritional aids of study were assessed in accordance with the tool developed by Close et al., a 9-step framework (Figure 4) to specifically evaluate performance nutrition research [14].

## 3. Results

### 3.1. Main Search

The literature search identified a total of 79 articles assessed for eligibility related to the selected descriptors. From these studies, 40 met the inclusion criteria, and 39 did not match the search descriptions. The age of basketball players for the studies ranged from adolescent (school and college) to adult for both sexes. In a decreasing order, the most evaluated supplement was: 11 studies of vitamins (VIT), 9 studies of caffeine (CAF), 6 studies about protein (PRO), 5 studies of carbohydrates (CHO), [4 CHO and 1 CHO + Creatine (CRE)], and a mix of other supplements conformed by 2 studies of nitrates (NIT), 2 studies of sodium bicarbonate (SB), 1 study of Eicosapentaenoic Acid (EPA),1 study of beta alanine (β-ALA), 1 study of cysteine (CYS), 1 study of glutamine (GLUT), 1 study of magnesium (MG) (Table 1). This systematic review classifies the ergo-nutritional aids in basketball to enhance game performance (Table 2) and to enhance recovery (Table 3).

### 3.2. Ergo-Nutritional Aids to Enhance Recovery in Basketball

#### 3.2.1. Carbohydrates and Proteins

A combination of 20 g of CHO (commercial CHO sports beverage) with 20 g CRE for 7 days was reported [70] to have a positive effect on recovery markers when administered to men. Ho et al. [67] found a positive association between a PRO supplementation strategy (6.25 kcal/kg high-PRO, 36% PRO in total calorie) and cerebral oxygenation during exercise, enhancing recovery. The short-term recovery effects of 20 g PRO supplementation also yielded consistent results in combination with 40 g of oligosaccharides eaten half an hour before going to bed [68]. In contrast, recovery markers (IL-10) were not optimized after orally supplementation of 3.2 g bovine colostrum twice a day for 24 weeks [49].

#### 3.2.2. Vitamins

A potentially large and significant effect of VIT E was found on recovery markers (total antioxidant status, protein S100B, acetylcholinesterase), when an absolute dosage ranging from 200 to 268 mg was administrated in non-elite players [56,58,59,60,61]. These findings were also consistent with a lower dosage of VIT E (150 mg) in a combination of VIT C (500 mg) [57] and when combined 268 mg VIT E with 2 g of EPA [56]. An intensified dosage of VIT E boosted to 600 mg and in combination with 1000 mg VIT C, and 8 mg beta-carotene over 32 days, ameliorate oxidative stress and therefore improving recovery markers (testosterone/cortisol, LDH), in professional basketball players during daily training [77]. Regarding VIT D, the effects of cholecalciferol supplementation on VIT D status was studied by Sekel et al. [41] in a mixed sample of female and basketball players. Neither 5000 IU/day nor 10,000 IU/day show significant improvements on recovery markers (25 OH D status); however, a larger dosage (10,000 IU/day) was reported to have protective effects. Finally, a descriptive study published in 1961 [62] reported that multivitamin supplementation improves the health of adolescent students, minimizing the number of occurrences and duration of colds.

#### 3.2.3. Others

One study performed a magnesium supplementation of 400 mg/day for an entire season [76]. It showed a positive association with recovery markers (ALD, ALT, and CK) in young basketball players. The potential effects of glutamine supplementation [75] in recovery biomarkers (AST, lymphocytes, and ACTH), revealed hormonal and white blood cell homeostasis. Nitrate consumption show a likely significative effect on recovery (TAS and plasma polyphenol levels) [50] when administrated as 200 g purple sweet potato. Finally, one study [56] investigated EPA’s potential to reduce the levels of pro-inflammatory markers and thus enhance recovery. The intake of 2 g for 6 weeks was associated with an enhancement on serum malondialdehyde levels. A single study [74] carried out following a 0.5 g/day of L-cysteine was associated with protective effect on recovery markers (CK, DNA oxidative damage, LDH, total antioxidant status). CRE supplementation was found in one study [70], showing a statistically improvement on recovery markers when following a guide of 20 g during 7 days and later 2 g/day.

### 3.3. Ergo-Nutritional Aids to Enhance on-Court Performance in Basketball

#### 3.3.1. Carbohydrates or Proteins

Hydration with only water versus hydration with an amount of CHO (commercial CHO electrolyte sports beverage) versus fluid restriction during an exercise was analyzed in three studies [35,36,69]. An impairment in general performance was found in the fluid restriction (dehydrated) groups; however, differences between a CHO-free solution (water) or a CHO-electrolyte sports beverage had a null to small effect on anaerobic or skill performance outputs. The intake of 24 g whey PRO or casein [47,48] for 8 weeks and immediately prior to and following training showed a remarkable improvement in anaerobic performance and body composition. However, no differences were found among them [47], yielding the same results.

#### 3.3.2. Caffeine

The CAF was tested in nine studies with a relative dosage ranging from 3 to 6 mg·kg^−1^. The measured effects of 3 mg·kg^−1^ supplementation 60–75 min before exercise and following a protocol test show small-moderate [43,64,66] to strong [63] improvement with regard to anaerobic performance, but only one study observed a notably enhancement in skill performance [65]. Regarding aerobic performance, three studies [43,44,66], analyzed its efficiency, but none of them offered advantages compared to control groups. Wellness was also evaluated, showing the opposite findings. Whereas Stojanovic et al. [64] found worse results due to the adverse side effects, others [43,44] found a special improvement in the feeling of vigorousness and energy. This opposition among benefits and adverse side effects was also reported by Puente et al. [65], who studied the effects of 3 mg·kg^−1^ of CAF in the protocol-simulated game but not contrasting variables. Lastly, a novel idea introduced by Stojanovic [63] has theorized that circadian biology may play a time-effect role, suggesting that improvements occur when is ingested in the morning but not in the evening. The intake of 6 mg·kg^−1^ following a protocol test showed a stronger improvement with regard to anaerobic performance [44,45] and wellness [44] considering feelings of vigorousness and energy as part of it; however, aerobic, external load, and skill performance did not show statistical improvements in both protocol- or match-simulated analyses.

#### 3.3.3. Others

Finally, buffer capacity was analyzed in three studies [71,72,73], with the opposite results. Whereas 0.2 g/kg SB administered 20 and 90 min before the exercise showed an enhancement in skill and anaerobic performance [71,72], β-ALA supplementation (6.4 g/day for 6 weeks) did not show the same results [73]. In addition, anaerobic performance shows a small-moderate improvement with SB administration [71,72] but this effect was not achieved with β-ALA [73]. The role of NIT (a commercial 140 mL beetroot juice, 12.8 mmol NO_3_) on anaerobic performance and external load was studied both following a protocol test and in a friendly match without statistical improvement in these variables [51].

## 4. Discussion

In this study, we aimed to review and evaluate the current scientific knowledge about the effect of ergo-nutritional supplements on basketball performance. The results of this systematic review have shown that the effective dose of CAF to enhance anaerobic performance and the feeling of vigorousness and energy ranges from 3 to 6 mg·kg^−1^, showing more positive effects when is supplemented 60–75 min before exercise in the morning and in a test-based task. On the other hand, vitamin E (ranging from 200 to 268 mg), vitamin D (10,000 IU) and EPA (2 g) may have a potential role in recovery and wellness. Despite the need for future studies, certain nutritional supplements may have promising advantages for basketball (long-term supplementation of nitrates for recovery), whereas others (β-alanine, sodium bicarbonate, acute nitrate supplementation) might theoretically be regarded as not interesting for basketball, or even not recommended by the World Anti-Doping Agency (WADA), as in the case of bovine colostrum (https://www.wada-ama.org/en/questions-answers/prohibited-list-qa) (last accessed on 4 January 2022). The main results indicate that some studies have found the opposite results, so it is essential to discuss each according to the demands of the players, in search of a strategy of personalized supplementation.

### 4.1. Ergo-Nutritional Aids to Enhance Recovery in Basketball

#### 4.1.1. Carbohydrates or Proteins

It is well known that CHO plays a key role in sports performance and fueling team sports such as basketball [8,37,78], promoting recovery [5,79] and regulating training adaptations [18,80]. There is a widespread variety of CHO-rich foods (rice, pasta, bread, fruits and vegetables, etc.), but the amount, type, timing and frequency of ingestion are critical concerns for sports nutrition [78,79,80,81] to prepare and recover athletes for the work required [18]. Basketball is a very demanding game with a short time to recover between games [4]; thus, having strong knowledge [26] about CHO fueling strategies may lead to a clear competitive advantage [5]. This review reaffirms CHO’s role in the recovery process [78], so when diet cannot cover these requirements, it may be recommendable support it with some ergogenic aids such as bars, gels, drinks, or similar until this recommendation is fulfilled. Our findings also support that a lower dosage can be used in a combination with an amount of 20 g of CRE [70,82] or a source (~20 g) of high-quality PRO [27,68].

The provision of PRO to retain muscle degradation and enhance reparation is strongly underpinned and established as a fundamental pillar in sports nutrition [83,84,85]. Some food sources of dietary PRO are meat, fish and seafood, eggs, dairy products, nuts, legumes and beans, and isolated forms of PRO can be used when appetite is suppressed (after high intensity exercise) or to achieve a high protein intake in the diet [8]. Our review supports that ingesting bolus from ~25 g PRO supplement immediately prior to exercise, and before bedtime, has a short-term positive effect on recovery markers (hemoglobin, red blood cell count, hematocrit, and mean corpuscular volume). Concerning the type of PRO, bovine colostrum and casein showed no significant differences with respect to whey PRO. Moreover, nowadays, bovine colostrum is considered a product not recommended by WADA (https://www.wada-ama.org/en/questions-answers/prohibited-list-qa) (last accessed 4 January 2022). Future studies should also look closely different types of PRO, including plant PRO.

#### 4.1.2. Vitamins

Oxidative stress is a natural process experienced by the basketball player as a result of the high intensity of the game [1]. This leads to the generation of free radicals, which are related to harmful effects on proteins, DNA and lipids, resulting in muscular fatigue and an increased risk of injury or some pathology [1]. Our review points out that the main purpose of vitamin supplementation is to achieve an antioxidant effect, to mitigate these negative effects. An absolute dosage of VIT E ranging from 200 to 268 mg seems to be effective in enhancing recovery [56,57,58,59,60,61]. However, these results should be treated with caution and consider some risk of bias, given that VIT E deficiency is relatively common in youth population mainly due to rapid growth [86]. Additionally, interest in VIT D has shown a powerful growth in scientific production in recent years [87,88,89] and also in basketball [90]. In our review, we found only one intervention theorizing a protective effect of 10,000 IU/day supplementation [41]. As no evidence-based consensus exists for VIT D supplementation, good nutrition and diet is nowadays the most important strategy to prevent the alarming proportion of basketball players with VIT D deficiency [87]. As basketball players normally train indoors, their exposition to the sun is limited [87]. This fact becomes especially critical for black players who are at higher risk of having insufficient plasma levels of this VIT [8] and in all athletes, during the winter months, when the human body has its lowest levels of VIT D. Thus, future studies should analyze deeply this topic and take into account emergent new basketball modalities such as 3 × 3 where the game can be played outdoors.

#### 4.1.3. Others

Many basketball actions require eccentric movements, triggering an elevated acute effect [91] and chronic muscle damage [92]. In medicine science, the anti-inflammatory potential of polyunsaturated fatty acids (PUFA) such as omega 3 and 6 [8] is well known. In our systematic review, we found one study [56] describing a positive association of 2 g EPA for 6 weeks with a reduction in the levels of pro-inflammatory markers and, consequently, recovery. Finally, although cysteine shows a protective effect on recovery markers [74], these results should be taken with great caution and expand the number of studies on this topic, due the existing concerns about its side effects with high doses [93]. Regarding glutamine supplementation, there is an emergent popularity in exploring its potential for sports performance, due its biological functions (anabolic properties, energy production, protein regulation, buffer properties, etc.) [75,94]. Particularly, glutamine supplementation aims to promote immunity and ensure a quicker recovery [95]. One the one hand, glutamine is the main fuel for immune cells; however, its availability decreases as a consequence of the intensity of the game [75]. On the other hand, glutamine plays a key role in glycogen synthesis, favoring glycogen restoration [94]. In addition to these ideas, as the game of basketball generates an inflammatory response as a consequence of the many eccentric movements, Córdova-Martínez and their colleges [75] investigated the potential role of glutamine supplementation over muscle damage, finding a positive association between 6 g/day of glutamine for 20 days. Finally, the role of magnesium (transporter, energetic metabolism, and relaxation/contraction) over muscular performance explains the interest it has received as an ergogenic aid given that high-intensity exercise may lead to hypomagnesemia, resulting in muscle fatigue [76]. A cross-sectional study was conducted to analyze the effects of 400 mg/day magnesium supplementation, showing a positive association with recovery markers (aldolase, alanine aminotransferase, and creatine kinase) in young basketball players [76]. However, as there is a lack of evidence on magnesium as supplementation [96], more studies are necessary to establish if these findings could be considered as eventual or really have a considerable potential effect on performance and recovery in team sports such as basketball [5].

### 4.2. Ergo-Nutritional Aids to Enhance on-Court Performance in Basketball

#### 4.2.1. Carbohydrate or Protein

The CHO are the main energetic fuel for basketball [8], allowing players to perform explosive and repeated movements [3]. However, glycogen storage is unlimited, so players, must replenish it efficiently (amount, type and timing) before and after the game. Three studies [35,36,69] have analyzed the effects of a small amount of CHO consumption in beverage form. No differences were observed in comparison with the control group (only water). However, beyond the ingestion of CHO during exercise, a promising future on CHO mouth rising [97] and modified CHO [98] may open future research lines in their role over central and peripheral function [78] with basketball players.

The role of PRO in basketball players is also crucial, minimizing protein catabolism promoting protein synthesis, and eventually helping muscle glycogen resynthesize [8]. Our review highlights that an administration of ~25 g PRO supplementation immediately prior to exercise and before sleep, has a remarkable improvement on anaerobic performance and body composition, which is in line with a previous expert’s consensus statement [81].

#### 4.2.2. Caffeine

Caffeine is an alkaloid largely consumed around the world in forms such as tea, cocoa or coffee [65]. It is not only used by the athlete population in search of a better performance, but also by the general population as part of its gastronomic culture [32]. Caffeine is considered a legal, safe and effective substance with a large ergogenic potential in many performance outcomes [30,99,100]. The main results of our systematic review indicate that 3 to 6 mg·kg^−1^ ingested 60–75 min before exercise is an effective nutritional strategy to boost anaerobic performance in basketball and may have a potential effect on skill performance, whereas aerobic performance did not show statistically significant improvement. The feeling of vigorousness and energy lead to an improvement in wellness and perceived self-capacity [43]. In a recent systematic review about the ergo-nutritional effects of caffeine in basketball [101], Sen-tan et al. highlight the influence of genetics (C-allele metabolizers or AA homozygotes) over specific outcomes (Abalakov jump test outputs and perceived muscle power) based on the CYP1A2 activity, and the main registered side effects (insomnia). Therefore, it is critical to understand a player’s responses and elucidate potential discomforts during or after exercise [63]. Lastly, the ecological effect over performance outcomes and impact of circadian rhythm [102] must be take into account. The first finding described by Raya-Gonzalez [44] suggested that caffeine could be effective to improve physical performance during tests but with no meaningful effects on the activity completed during simulated basketball. Secondly, the theory proposed by Stojanovic [63] postulates that caffeine consumption during the morning is the most effective moment to use this ergogenic aid. In the case of basketball, future works should increase efforts describing possible differences between sexes, such as those that Mielgo-Ayuso et al. found in a recent systematic review [32], and also regarding genetics [102]. The form of use (capsule, drink, gum, mouth rising, etc.) [103] is another critical issue to understand possible differences due to formulation and in addition to develop new strategies for minimal adverse side secondary effects [101].

#### 4.2.3. Others

Regarding buffer-nutritional aids (sodium bicarbonate and β-alanine), our review describes contradictory results for basketball performance.

Although the promising effects of dietary nitrates on performance are described [104], our review found opposing findings, suggesting that basketball players could benefit from these effects with chronic and medium-long-term consumption, rather than acute intake.

Despite the large popularity of CRE supplementation [79], we only found one study carried out in basketball which support it role on recovery proposed by Roberts et al. [82]. A deeper understanding of CRE supplementation in basketball is needed to establish precisely the effective dose, load strategy, pre-evaluation of responders, possible adverse side effects, or potential differences between sexes, as a recent review describes in the context of soccer [33], and also new potential outcomes of CRE beyond muscle [105].

### 4.3. Limitations, Strengths, and Future Research Lines

This study presents some limitations. The main one is the scarcity of studies carried out in relation to nutritional supplementation in basketball players. Additionally, the power of the analysis was not enough to conduct a meta-analysis.

However, one important strength is that (to the best of our knowledge) this is the first systematic review describing which ergo-nutritional aids can be specifically useful in basketball to enhance on-court performance and recovery, boosting future studies and helping players and team staff to plan their ergo-nutritional aids wisely. This is critically important given that basketball players do not have enough nutritional knowledge to manage it on their own [26]. Thus, it should be noted that our systematic review should be taken as a first step to normalize study designs and categorize the main outcomes to get a big picture of the main effects of a nutritional supplementation intervention.

The long-term supplementation of nitrates for recovery and anaerobic performance should also be observed in future studies. On the other hand, it seems that some others (β-ALA, SB, acute NIT supplementation) might be theoretically considered as not of interest for basketballers or even banned (BC) by WADA. Finally, new research lines should focus on describing the potential exact effects and interactions of some nutritional supplements with strong scientific evidence, such as CRE, also considering sex differences.

## 5. Conclusions

Before using supplements, a well-planned, nutritious and varied diet should be developed in order to meet basketball player’s needs. Dietary screening and (where applicable) nutrition education programs should also be implemented prior to an ergo-nutritional intervention. In addition, this should also cause no impairments, and be implemented in search of a real ergogenic effect. If ergo-nutritional supplementation is needed in basketball, the results of this systematic review suggest that:

- The effective dose of CAF to enhance anaerobic performance and the feeling of vigorousness and energy, ranges from 3 to 6 mg·kg^−1^, showing more positive effects when is supplemented 60–75 min before exercise in the morning and in test-based task.
- To improve recovery, the management of PRO is a key factor, when following a ~0.5 g/kg strategy or 25 g PRO supplementation immediately prior to exercise and before bed time and, additionally, in combination with CHO (1 g PRO/kg with 1 g CHO/kg or 20 g of CHO with 20 g CRE for 7 days).
- To improve recovery and wellness, some nutritional supplements may have promising benefits for basketball. This is the case of Vitamin E (ranging from 200 to 268 mg), vitamin D (10,000 IU/day) and EPA (2 g). However, future studies are necessary.
- The form of use (capsules, liquid, pill, powder) and other organoleptic characteristics (such as flavor or texture) should be considered to achieve better adherence to the intervention.

## Figures and Tables

**Table 1:** Summary of the studies analyzed (grouped by ergo-nutritional aid).

**Authors**	**Population (Level and *n*-Size)**	**Study Design**	**Intervention (Form)**
**Vitamins**			
Sekel et al. (2020) [41]	College female and male (*n* = 20)	Quasi experimental	5000 IU/day VIT D (capsule)
Sekel et al. (2020) [41]	College female and male (*n* = 20)	Quasi experimental	10,000 IU/day VIT D (capsule)
Ghiasvand et al. 2010 [56]	Highly trained male (*n* = 9)	Double blind placebo control randomized	400 IU VIT E (soft gel)
Ghiasvand et al. (2010) [56]	Highly trained male (*n* = 8)	Double blind placebo control randomized	2 g EPA + 400 IU VIT E (soft gel)
Naziroǧlu et al. (2010) [57]	Elite male (*n* = 14)	Intervention control no placebo	VIT E 150 mg +VIT C 500 mg (gelatin capsule)
Schulpis et al. (2007) [58]	College male (*n* = 10)	Intervention	200 mg VIT E *
Schulpis et al. (2007) [59]	Adolescent male (*n* = 10)	Intervention control no placebo	200 mg VIT E *
Tsakiris et al. (2006) [60]	Adolescent male (*n* = 10)	Intervention control no placebo	200 mg VIT E *
Tsakiris et al. (2006) [61]	Adolescent male (*n* = 10)	Intervention control no placebo	200 mg VIT E *
Schröder et al. (2000) [25]	Elite male (*n* = 24)	Placebo no control	250 mg VIT C + 8 Mg VIT E + 150 mg VIT A (4/day/35/days) (capsule)
Barnes et al. (1961) [62]	Adolescent male and female (*n* = 26)	Intervention control no placebo	Not determined *
**Caffeine**			
Raya-González et al. (2021) [44]	Elite male (*n* = 14)	Double blind placebo control randomized	6 mg·kg^−1^(60 min before) (liquid form)
Stojanovic et al. (2021) [63]	Adolescent male (*n* = 11)	Double blind placebo control	3 mg·kg^−1^ (60 min before) (capsule)
Tan et al. (2020) [46]	Elite male (*n* = 12)	Randomized controlled trial	6 mg·kg^−1^ (60 min before) (CAF powder dissolved in water)
Stojanovic et al. (2019) [64]	Elite female (*n* = 10)	Double blind placebo control	3 mg·kg^−1^ (60 min before) (capsule)
Scanlan et al. (2019) [42]	Elite male (*n* = 11)	Double blind placebo control randomized	3 mg·kg^−1^ (60 min before) (capsule)
Puente et al. (2017) [65]	Elite male (*n* = 10)	Double blind placebo control randomized	3 mg·kg^−1^ (75 min before) (capsule)
Cheng-Feng (2016) [45]	College male (*n* = 15)	Double blind no control randomized	6 mg·kg^−1^ (60 min before) (capsule)
Abian-Vicen et al. (2014) [43]	College male (*n* = 16)	Double blind placebo control	3 mg·kg^−1^ (60 min before) (energy drink powder)
Tucker et al. (2013) [66]	Elite male (*n* = 5)	Placebo no control	3 mg·kg^−1^ (60 min before) (Tablet)
**Protein**			
Skarpa et al. (2020) [49]	Elite female (*n* = 20)	Intervention control no placebo	3.2 g BC twice a day for 24 weeks (capsules)
Feng-Ho et al. (2018) [67]	Elite male (*n* = 16)	Randomized counterbalanced	600 mL drink (6.25 kcal/kg; 36% PRO; 58% CHO; 6% FAT) (beverage)
Taylor et al. (2015) [48]	College female (*n* = 8)	Placebo no control	24 g (Whey PRO)/8 weeks/pre and post training (Powder)
Ronghui et al. (2015) [68]	College male (*n* = 5)	Intervention control no placebo	20 g (whey PRO) + OLI 40 g every two days (Powder)
Wilborn et al. (2013) [47]	College female and male (*n* = 20)	Double blind no control randomized	24 g (whey PRO)/8 weeks/pre and post training (Powder)
Wilborn et al. (2013) [47]	College female and male (*n* = 20)	Double blind no control randomized	24 g (casein PRO)/8 weeks/pre and post training (Powder)
**Carbohydrate**			
Carvalho-Bruno et al. (2011) [35]	Adolescent male (*n* = 20)	Crossover design placebo control	8% CHO (beverage)
Baker et al. (2007) [36]	Highly trained male (*n* = 17)	Intervention control placebo	6% CHO (beverage)
Dougherty et al. (2006) [69]	Adolescent (*n* = 15)	Double blind control placebo	6% CHO and 18.0 mmol·L^−1^ Na(beverage)
Shi et al. (2005) [70]	Highly trained male (*n* = 20)	Intervention control no placebo	Commercial drink (100 g/L) 500 mL/day(beverage)
Shi et al. (2005) [70]	Highly trained male (*n* = 20)	Intervention control no placebo	20 g CHO (beverage) + 20 CR (powder) for 7 days
**Nitrates**			
López-Samanes et al. (2020) [51]	Adolescent male (*n* = 12)	Double blind placebo control	140 mL beetroot juice (12.8 mmol NO_3_) (beverage)
Chang et al. (2007) [50]	Elite male (*n* = 6)	Intervention control no placebo	200 g/day sweet potato leaf (solid meal)
**Sodium Bicarbonate**			
Ansdell et al. (2020) [71]	Highly trained male (*n* = 10)	Placebo no control	0.2 g/kg (90 min before) + 0.2 g/kg (60 min before) (powder)
Gregg Afman et al. (2014) [72]	College male (*n* = 7)	Randomized counterbalanced	0.2 g/kg (90 min before) + 0.2 g/kg (20 min before) (powder)
**EPA**			
Ghiasvand et al. (2010) [56]	Highly trained male (*n* = 8)	Double blind placebo control randomized	2 g EPA (soft gel)
**Beta Alanine**			
Milioni et al. (2017) [73]	College male (*n* = 27)	Placebo no control	6.4 g/day (capsule)
**Cysteine**			
Tsakiris et al. (2006) [74]	College male (*n* = 10)	Intervention control no placebo	0.5 g 24 h^−1^ for 30 days (powder)
**Glutamine**			
Córdova-Martínez et al. (2021) [75]	Elite male (*n* = 12)	Double-blind, placebo-controlled trial	6 g/day (20 days) (capsule)
**Magnesium**			
Córdova Martínez et al. (2017) [76]	Elite male (*n* = 12)	Longitudinal no control no placebo	400 mg/day (complete season) *

Legend: Caffeine (CAF); Carbohydrate (CHO); Creatine (CRE); Protein (PRO); Vitamin (Vit); Eicosapentaenoic Acid (EPA); Beta Alanine (β-ALA); Cysteine (CYS); Glutamine (GLUT); Magnesium (MG); Nitrates (NIT); Sodium Bicarbonate (SB); International Units (IU); * Form of use not detailed.

**Table 2:** (1) Ergo-nutritional aids for the on-court performance (anaerobic and aerobic outcomes). (2) Ergo-nutritional aids for the on-court performance (strength and body composition).

**(1)**			
**Ergo-Nutritional Aid**	**Intervention Dose (Administration)**	**Outcome**	**Results**
CAF	3 mg·kg^−1^ (60–75 min before)	Sprint without ball (t)	→+++
	3–6 mg·kg^−1^ (60–75 min before)	Agility Run (m/s) CMJ (cm) COD Without Ball (t)	→++ →+++ ++
	6 mg·kg^−1^ (60–75 min before)	Work Done Above End-Test Power (Kj) RSA (t)	+ ++
β-ALA	6.4 g/day (6 weeks)	RSA (t) Peak Blood Lactate Concentration (mmol/L)	+ +
CHO	Beverage (6–8% CHO)	RSA (t) Skill performance	→ →
SB	0.2 g/kg (90 min before) + 0.2 g/kg (20 min before)	RSA (t) Peak Blood Lactate Concentration (mmol/L)	+ +
	0.2 g/kg (90 min before) + 0.2 g/kg (60 min before)	RSA (t) Skill Performance	→ →
PRO	Whey PRO 24 g/day (8 weeks)	Agility (m/s) CMJ (cm) Broad Jump (D)	++ ++ ++
	Casein PRO 24 g/day (8 weeks)	Agility (m/s) CMJ (cm) Broad Jump (D)	+ + +
	600 mL beverage (6.25 kcal/kg; PRO: 36%; CHO: 58%; fat: 6% in total calorie)	Sprint without ball (t)	→+++
	250 mL whole milk + 20 g whey PRO + 40 g oligosaccharides	Agility Run (m/s) CMJ (cm) COD without ball (m/s)	→++ →+++ ++
**(2)**			
**Ergo-Nutritional Aid**	**Intervention Dose (Administration)**	**Outcome**	**Results**
PRO	Whey PRO 24 g/day/8 weeks	Strength upper body (1RM-Kg) Strength lower body (1RM-Kg) Body fat (%) Lean mass (kg)	++ ++ ++
	Casein PRO 24 g/day/8 weeks	Strength upper body (1RM-Kg) Strength lower body (1RM-Kg) Body fat (%) Lean mass (Kg)	+ + +

Legend: Caffeine (CAF); Beta Alanine (β-ALA); Carbohydrate (CHO); Sodium Bicarbonate (SB); Protein (PRO); Counter Movement Jump (CMJ); Change of Direction (COD); Repeated Sprint Ability (RSA). Neutral effect (→); Positive Effect (+). Legend: Protein (PRO); Repetition Maximum (RM).

**Table 3:** Ergo-nutritional aids for game recovery.

Ergo-Nutritional Aid	Intervention Dose (Administration)	Outcome	Results
CRE	20 g/day (7 days) + 2 g/d	Blood Urine Nitrogen (mmol/L)	+
CRE+CHO (OLI)	20 g/day CRE (7 days) + 20 CHO/day	Blood Urine Nitrogen (mmol/L))	+
		CK (U/L)	+
EPA	2 g/day (6 weeks)	MDA (nmol/L)	+
GLUT	6 g/day (20 days)	AST (U/L) Lymphocytes (%)	+ +
MG	400 mg/day (complete season)	ALD (U/L)	+
		ALT (U/L)	+
		CK (U/L)	+
NIT	200 g/day sweet potato leaf	Total Antioxidant Status (mmol/L) Plasma polyphenol levels mg GAE/dL)	+ +
CHO (OLI)	500 mL beverage (100 g/mL)	Blood Urine Nitrogen (mmol/L)	+
		CK (U/L)	+
CYS	0.5 g/day (one month)	CK (U/L)	+
		DNA Oxidative Damage (8-OHdG (ng mL^−1^))	+
		LDH (U/L)	+
		Total Antioxidant Status (mmol/L)	+
PRO + CHO	250 mL whole milk + 20 g whey PRO + 40 g oligosaccharides (once every two days)	Hemoglobin (g/dl) RBCC (cells/mcL) Hematocrit (%) MCV (fl)	+ + + +
VIT D	10,000 IU/day (5 months)	25(OH)D Status	→
	5000 IU/day (5 months)	25(OH)D Status	-
VIT E + EPA	400 IU VIT E+ 2 g EPA (6 weeks)	Glutathione Reductase (u/L)	+
		TNF-α(pg/mL)	+
		IL-2 (pg(mL)	+
VIT C + E	500 mg VIT C + 150 mg VIT E/Day (One Month)	GSH-Px (IU/g protein)	+
		LP (umol/g protein)	+
VIT C + E + A	250 mg VIT C + 8 mg VIT E + 150 mg VIT A (4 Times Day/35 Days)	Testosterone/Cortisol (pg mL^−1^)	→
		LDH (U/L)	→
VIT E	200 mg/Day (One Month)	Total Antioxidant Status (mmol/L)	+++
		Protein S100B (ug/L)	+
		Acetylcholinesterase (ΔOD/min/mg protein)	+
		DA, A, NA (pmol^−1^)	+
	400 IU (6 Weeks)	Catalase Activity (U/mg)	+
BC	Define As “Ilegal” (WADA)		

Legend: Creatine (CRE); Oligosaccharide (OLI); Eicosapentaenoic Acid (EPA); Glutamine (GLUT); Magnesium (MG); Nitrates (NIT); Cysteine (CYS); Carbohydrate (CHO), Vitamin (VIT); Bovine Colostrum (BC); International Units (IU); World Anti-Doping Agency (WADA); Creatine Kinase (CK); Serum Malondialdehyde level (MDA); Aldolase (ALD); Aspartate Aminotransferase (AST); Alanine Aminotransferase (ALT); Creatine Kinase (CK); Lactate Dehydrogenase (LDH); Red Blood Cell Count (RBCC); Mean Corpuscular Volume (MCV); Hidroxi (OH); Tumor Necrosis Factor-alpha (TNF-α); Interleukin-2 (IL-2) Glutathione Peroxidase (GSH-Px); Lipid Peroxidation (LP); Dopamine (DA); Adrenaline (A); Noradrenaline (NA); Neutral effect (→); Positive Effect (+); Negative Effect (-).

## Data Availability

Not applicable.
